# What we actually know about the pathogenicity of *Bacteroides pyogenes*

**DOI:** 10.1007/s00430-021-00709-2

**Published:** 2021-05-02

**Authors:** Anna Majewska, Marta Kierzkowska, Dariusz Kawecki

**Affiliations:** Department of Medical Microbiology, Medical University of Warsaw, Chalubinskiego 5 Str, 02-004 Warsaw, Poland

**Keywords:** Anaerobic infection, *Bacteroides pyogenes*, MALDTI-TOF MS, Penicillin resistance, Zoonotic infection, 16S rRNA

## Abstract

The aim of the study was to evaluate the pathogenic potential of *Bacteroides pyogenes,* rarely identified in clinical laboratories anaerobic bacteria. To increase the knowledge about this poorly understood anaerobic microorganism, the study also includes cases of infections described so far in the literature. Only the use of 16S rRNA sequencing and mass spectrometry technique allowed the identification of *B. pyogenes* from clinical specimens. We reported 13 severe human infections caused by *B. pyogenes*. Bacteria were cultured from the wound after biting by animals, chronic infections within the oral cavity, from patients with histologically or radiological proven osteomyelitis, surgical site infection, and from urine sample collected after a urological procedure. Most (9/13) of the patients required hospitalization. Almost 70% of them needed urgent admission via the emergency room. Two inpatients due to a life-threatening condition were admitted to the intensive care unit. Almost 50% of isolates were resistant to penicillin. All resistant to penicillin strains were isolated from skin and mucous membrane infections.

## Introduction

Anaerobic bacteria are important human pathogens and are involved in endogenous, opportunistic infections of every part of the body including skin and subcutaneous tissues, mainly because of a disruption in tissue barriers. Although anaerobes were first discovered and identified in the 60. of the XIX century the knowledge about their pathogenicity in humans is still incomplete, but thanks to new microbiological techniques it is systematically expanded. Nevertheless, anaerobic infections are the most overlooked of all bacterial infections. Of all known anaerobes *Bacteroides* are the most often isolated from clinical specimens [1–4]. To date, *Bacteroides fragilis* is regarded as the most virulent. Other clinically relevant species are: *B*. *ovatus, B*. *thetaiotaomicron*, *B. vulgatus, and Parabacteroides distasonis* [3]*.* The use of 16S rRNA sequencing and mass spectrometry technique allowed the identification of other *Bacteroides*, rarely described so far in the medical literature. One of them is *B. pyogenes* [5–8]*.*

The taxonomy of the genus *Bacteroides* has undergone significant changes. This also applies to *Bacteroides pyogenes*. 16S rRNA and *hsp60* gene sequencing were used for the classification and establishment of phylogenetic relationships. Sakamoto et al*.* concluded that *B. suis* and *B. tectus* are heterotypic synonyms of *B. pyogenes* [9]*.* Four years later the whole-genome sequencing of these three strains was expected to provide new information. Analyses of genomes revealed the diversification of *B. pyogene*s strains isolated from different animals [10]. Further analysis will improve the understanding of this species.

Presently it is known that *B. pyogenes* is an obligately anaerobic Gram-negative bacilli (AGNB), non-motile, non-pigment-forming, non-spore-forming, produce succinic and acetic acids. *B. pyogenes* is a component of the natural microbiota of the animal’s (dogs and cats) oral cavity [11, 12]. *B. pyogenes* was first isolated from abscesses and feces of pigs in the 1980s, then from the uterus of metritis [11, 13, 14] and from the horse's wound [15]. It has been reported that *B. pyogenes* is a human pathogen, as well. The findings of 10 patients with *B. pyogenes* infection, described so far in the literature, are presented in Table 1. All isolates were identified by 16S rRNA sequencing or by Bruker matrix-assisted laser desorption ionization-time of flight mass spectrometry (MALDTI-TOF MS) [5–8]. To date, no information on the biology and pathogenicity of this species has been published.

**Table 1:** Clinical characteristics of *Bacteroides pyogenes* infections described in the literature

Clinical characteristics	Specimen	Etiology	Ref
Bacteremia secondary to cat bite	Smear from the bite wound	*Bacteroides pyogenes, Pasteurella multocida*	[5]
	Blood	*Bacteroides pyogenes*	
Bacteremia secondary to liver abscess; no history of an animal exposure	Blood	*Bacteroides pyogenes*	[8]
	Aspiration of the liver abscess	*Bacteroides pyogenes, Klebsiella pneumoniae*	
Prosthetic joint infection	Replaced polyethylene	*Bacteroides pyogenes, Peptostreptococcus canis*	[6]
	Sonicated fluid of the prosthesis	*Bacteroides pyogenes*	
3 cases of dog bites	Smear from the wound	*Bacteroides pyogenes*	[7]
		*Bacteroides pyogenes, Pasteurella canis* *Pasteurella stomatis, Staphylococcus pseudintermedius*	
		*Bacteroides pyogenes, Pasteurella dogmatis*	
3 cases of cat bites		*Bacteroides pyogenes* (2 cases)	
		*Bacteroides pyogenes, Pasteurella multocida*	
Trauma; amputation, revision of a stump	Smear from the wound	*Bacteroides pyogenes, Staphylococcus aureus*	

The objective of the study is to evaluate the pathogenic potential of *B. pyogenes by presenting* the cases of infections observed at our laboratory. Additionally, we reviewed the literature to summarize the experience with *B. pyogenes* infectious and to clarify, in the light of the current knowledge, certain clinical, and therapeutic issues related to this anaerobic bacteria.

## Materials and methods

A retrospective study was conducted including infected patients from whom *B. pyogenes* has been isolated from the site of infection. Bacterial isolates were collected during a routine microbiological examination of samples taken from adult patients treated at the Infant Jesus Teaching Hospital in Warsaw, Poland. In the period of 2013–2020 fifteen strains of *B. pyogenes* from thirteen patients were isolated.

The bacterial strains were cultured from the following specimens: pus (1), swab from an abscess (1) or wound (7), swab from the alveolar jaw (1), intraoperatively taken tissue (2), transtracheal aspirate (1), bile (1), urine (1). The specimens were cultured on bacteriological (Blood agar, MacConkey agar, Chocolate agar) and mycological (Sabouraud agar) media at 37 °C. To isolate anaerobic bacteria each sample was cultured on Schaedler agar with 5% sheep blood and vitamin K1, incubated for 48 h in anaerobic conditions (Genbox anaer, bioMérieux). All media were provided by bioMérieux, France. Presumptive identification using Gram staining preceded identification by MALDI-TOF MS technique in the Vitek MS v3.0 system (bioMérieux). All isolates were identified with an acceptable confidence value of 99.9%.

Drug susceptibility assessment was performed by the Etest method (bioMérieux) using strips impregnated, commonly used to treat anaerobic infections antibiotics: benzylpenicillin, amoxicillin with clavulanic acid, imipenem, clindamycin and metronidazole. The antibiogram interpretation was conducted in accordance with The European Committee on Antimicrobial Susceptibility Testing [16]. All samples were collected as part of routine hospitals surveillance.

## Results

This retrospective study involved 13 patients (5 male and 8 female) at the age from 35 to 89 (average 64.2) years old. Four patients were treated at the hospital outpatient clinics. Nine (69%) required hospitalization. Six of them (67%) because of a serious infection needed urgent admission via the emergency room. Two inpatients (22%) were admitted to the intensive care unit (ICU) due to a life-threatening condition. Clinical details of patients and microbiological findings were shown in Table 2 and Table 3, respectively.

**Table 2:** Clinical characteristic of patients: diagnosis, diagnostic procedures and antibiotic therapy

Dep	Patient no age/sex	Clinical presentation/diagnosis	CC	Surgical procedures	ED	ICU	Antibiotic therapy	Time in hospital	Specimen/sample/organ	Diagnostic procedures
Department of Cranio-Maxillofacial Surgery	1 67/M	Extensive, chronic soft tissue necrosis within oral cavity, osteomyelitis of the mandible bone *Carcinoma of the lower lip with reconstruction plate and radiation therapy (3 years earlier)	*ONCO IMM IMP	Surgical debridement, sequestromy Removal of reconstruction plate	No	Yes	gentamicin, ampicillin	28 days	Pus from an abscess	Microbiology; PB culture
									Mandibular bone fragment	Histopatology
									Head	CT
	2 70/M	Odontogenic infection, chronic maxillary sinusitis (left) Osteomyelitis of the jaw bone	–	Surgical debridement, drainage	No	No	clindamycin	3 days	Swab from the alveolar jaw	Microbiology; PB culture
									Swab from sinus	Microbiology; negative culture
									Bone fragment	Histopatology
	3 84/F	Phlegmon on the skin (neck and anterior chest wall), necrotic lesions with fistulas, primary infection in the oral cavity	–	Surgical debridement, drainage	Yes	No	metronidazole, vancomycin	36 days	Swab from wound	Microbiology; PB culture
									Chest	X-ray, CT
	4 35/M	Trauma; multiple fracture of mandibular bones with displacement, subcutaneous emphysema, necrotic inflammation of the submandibular and pharynx area	–	Surgical debridement, drainage, intubation	Yes	Yes	ampicillin, clindamycin (7 days) then ceftriaxone, metronidazole	23 days	Intraoperatively taken tissue	Microbiology; PB culture
									Transtracheal aspirate	Microbiology; MB culture
									Head	X-ray, CT
Department of General Surgery	5 67/M	Forefoot necrosis, edema, surgical site infection (amputation right foot, II and III finger), bubbles filled with gas, osteomyelitis	DM	Repeated surgical debridement	Yes	No	metronidazole, clindamycin	repeated hospital admissions in short intervals	Intraoperatively taken tissues	Microbiology; PB culture
									Forefoot	X-ray
	6 73/F	Cholecystolithiasis	–	–	Yes	No	ceftriaxone	10 days	Bile	Microbiology; PB culture
	7 89/F	Cat bite, phlegmon (right hand)	–	Surgical debridement drainage	No	No	amoxicillin/clavulanic acid, azithromycin	3 days	Swab from abscess	Microbiology; MB culture
Department of Urology	8 72/F	Bladder carcinoma	ONCO DM	Transurethral resection of a bladder tumor	Yes	No	cefuroxime (intraoperative)	22 days	Urine sample collected by the ureter catheter	Microbiology; PB culture CT
Department of Internal Medicine	9 79/F	Subcutaneous tissue inflammation, phlegmon (right foot)	–	Surgical debridement	Yes	No	ND	61 days	Swab from wound	Microbiology; MB culture 29 days later PB culture
									Right foot	X-ray
Orthopedic ambulatory	10 70/F	Dog bite	ND	ND	No	No	ND	N/A	Swab from wound	Microbiology; PB culture
	11 35/F	Dog bite	ND	ND	No	No	ND	N/A	Wound swab (finger II, left hand)	Microbiology; PB culture
Surgical ambulatory	12 39/F	Wound	ND	ND	No	No	ND	N/A	Swab from wound	Microbiology; PB culture
	13 55/M	Wound	ND	ND	No	No	ND	N/A	Swab from wound	Microbiology; PB culture

*Dep* department, *CC* co-occurring, *M* male, *F* female, *ED* Emergency Department, *ICU* Intensive Care Unit, *ONCO* oncologic history, *IMM* immunosuppression history, *IMP* artificial material, implant, *DM* diabetes mellitus, *PB* polybacterial infection, *MB* monobacterial infection, *CT* computer tomography, *ND* no date, *N/A* not applicable

**Table 3:** Microbiological characteristic of patients: specimens tested, co-pathogens isolated, *B. pyogenes* antimicrobial susceptibility

Patient No	Specimen	Microbiological results				
		*B. pyogenes* susceptibility^a^			Co-isolated bacteria	
			PEN	CLI	aerobic bacteria	anaerobic bacteria
1	Pus/swab from an abscess	*B. pyogenes*	R	R	*Eikenella corrodens* *Enterococcus faecalis* *Staphylococcus pneumoniae*	
2	Swab from the alveolar jaw	*B. pyogenes*	R	S	*Enterococcus faecalis* *Streptococcus parasanquinis*	
3	Wound swab	*B. pyogenes*	R	S	*Staphylococcus epidermidis*	-
4	Intraoperatively taken tissue	*B. pyogenes*	S	S	*Streptococcus anginosus*	*Fusobacterium nucleatum*
	Transtracheal aspirate	*B. pyogenes* > 10^5^ CFU_/mL_	S	S	*–*	–
5	Intraoperatively taken tissue	*B. pyogenes*	S	S	*Proteus mirabilis*	*Finegoldia magna*
6	Bile	*B. pyogenes*	S	S	*Escherichia coli*	*Clostridium perfringens*
7	Pus/swab from an abscess	*B. pyogenes*	R	S	*–*	*–*
8	Urine (ureteral catheter)	*B. pyogenes* 10^3^ CFU_/mL_	S	S	–	*Veillonella atypica*
9	Wound swab	*B. pyogenes*	S	S	*–*	*–*
		*B. pyogenes*	R	S	*Enterococcus faecalis* HLAR *Pseudomonas aeruginosa* *Alcaligens faecalis* *Staphylococcus aureus* MRSA	*Parvimonas micra*
10	Wound swab	*B. pyogenes*	S	S	*Pasteurella multocida*	*Fusobacterium nucleatum*
11	Wound swab	*B. pyogenes*	R	S	–	*Peptostreptococcus harei * *Cutibacterium acnes*
12	Wound swab	*B. pyogenes*	R	S	*Staphylococcus aureus*	*Fusobacterium nucleatum*
13	Wound swab	*B. pyogenes*	S	S	*Pasteurella canis*	*–*

*R* resistant, *S* susceptible, *PEN* penicillin, *CLI* clindamycin, *CFU* colony forming unit

^a^All *B. pyogenes* isolates were susceptible to amoxicillin/clavulanic acid, imipenem, metronidazole

Analysis of patients hospitalized in the Department of Cranio-Maxillofacial Surgery (Patient No.,,1”to,,4”) suggests that the human oral cavity may be a source of *B. pyogenes* (resident microbiota or transient colonization) and the presence of additional factors such as: trauma, including surgical interventions, presence of an artificial implant, cancer, chronic inflammation, and advanced age can predispose to deep tissue infections and even osteomyelitis. Infections with the participation of *B. pyogenes* were chronic and had polybacterial nature; one to three species of other bacteria from the single sample were co-cultured. All patients required surgical debridement of wounds, three necessitated long-term hospitalization (23–36 days). Analysis of the two patients’ history showed the involvement of *B. pyogenes* in histologically confirmed osteomyelitis (mandible and jaw; Patients No.,,1” and,,2”, respectively). In Patient No.,,4” besides intraoperatively taken sample, aspirate during endotracheal intubation was taken and merely *B. pyogenes* was cultured (> 10^5^ CFU/ml; CFU; colony forming units). In this patient, extensive infection with gas-filled bubbles and necrotic inflammation in the submandibular and pharyngeal area was accompanied by fluid in both pleural cavities. This patient required mechanical ventilation support and was hospitalized in ICU.

Three patients were admitted to the Department of General Surgery. In patient No.,,5” with diabetes mellitus surgical site infection (SSI) was diagnosed. Within 73 days after amputation surgical debridement of the wound have been performed three times. Patient required repeated in the short intervals’ hospitalization. Forefoot necrosis was finally diagnosed. *B. pyogenes, Finegoldia magna* and *Proteus mirabilis* were isolated from an intraoperative taken specimen. Osteomyelitis was confirmed by radiological examination.

*B. pyogenes* was isolated from a bile sample (Patient No.,,6”; 73-year-old woman with cholecystolithiasis) and from a urine sample collected from the 72-year-old woman hospitalized in the Department of Urology (Patient No.,,8”). She had transurethral resection of a bladder tumor (TURBT). *B. pyogenes*, to our knowledge, have never been isolated from such specimens before.

Bacteria were also isolated from the wound after biting by domestic animals (cat—Patient No.,,7″, dog—Patients No.,,10″ and,,11″). In one other Patient (No.,,13” with incomplete medical history) *B. pyogenes* was isolated from wound together with *Pasteurella canis*, what suggest zoonotic infection. In other patient with infected wound (Patient,,12”) we do not confirm any contact with animals.

Patient No.,,9″ (79—year old women) deserves special attention because of the presence of an acute, purulent inflammation on the skin and subcutaneous tissue infections. From the smear of the wound only *B. pyogenes* (monobacterial infection) was isolated, but 29 days later, probable because of long-term hospitalization (61 days), other five species were co-cultured; including methicillin-resistant *Staphylococcus aureus* (MRSA) and *Enterococcus faecalis* with high-level aminoglycoside resistance (HLAR).

Detailed characteristics of 4 patients (No. from,,10” to,,13”) were not available for us because they were treated at the hospital outpatient clinics.

We recorded that six of the seven inpatients with skin and mucous membranes injury infection were chronic, and five of the seven patients required long-term hospitalization (from 22 to 61 days). Nine out of eleven skin and mucous membranes infection was polybacterial (Table 3).

All of the isolated *B. pyogenes* strains were susceptible to amoxicillin with clavulanic acid, imipenem, and metronidazole. Overall, 7 of the isolates were resistant to penicillin, 1 isolate was resistant to clindamycin. We noticed that all penicillin-resistant *B. pyogenes* strains were isolated from surface infections (skin and mucous membranes). Isolates from bile, urine, and intraoperatively taken samples were susceptible to this antibiotic. All patients received empirical treatment covering the spectrum of cultured bacteria.

## Discussion

Applications of MALDI-TOF mass spectrometry allowed to identify *B. pyogenes* in clinical samples. To our knowledge, only ten cases of infection with this anaerobic species have been described so far in medical literature. In a retrospective analysis of patients with microbiologically proven *B. pyogenes* infection, we confirmed the reports of other researchers that *B. pyogenes* participate in infections after a dog and cat bites. Such infections were monobacterial or polybacterial. It is known that associations between many microorganisms often complicate the treatment of infections [5, 7].

The new observation is the presence of *B. pyogenes* in the urine sample collected by the ureter catheter. Bacteria in a titer of 10^3^ CFU/mL were isolated together with other anaerobic bacteria—*Veillonella atypica*. Anaerobes are treated as rare uropathogens (< 1%). *Veillonella* usually resides on the oral mucosa and genital tract. However, the case report published by Berenger et al. proves participation of anaerobic bacteria in cystitis and pyelonephritis, especially in patients with a history of catheterization and/or instrumentation [17]. This information can be obtained only by using sensitive methods of bacterial identification, e.g., MALDI–TOF or 16S rRNA sequencing.

*B. pyogenes* was also isolated from the bile sample. In the clinical case reported by Park JE et al. bacteria was responsible for liver abscess [8]. Both cases concerned women over 70 years of age with cholecystitis and radiolucent stones.

In the presented study, we noticed that *B. pyogenes* causes chronic oral infections. These infections were spread to surrounding tissues and cause necrosis, osteomyelitis, and fistula.

Osteitis and osteomyelitis are serious complications but based on the analysis of the literature it seems that anaerobic etiology is uncommon. In fact, however, many anaerobic infections are presumably underestimated because of difficulties with isolation and identification [4, 18]. Anaerobes are notable in osteomyelitis of cranial and facial bones which is usually caused by oral microbiome that spread from a contiguous soft-tissue source, from sinus, or dental infection [3]. We have observed that *B. pyogenes* participates in this pathology. Published reports indicate that *Bacteroides* spp*.* seldom leads to surgical site infection (SSI), especially if it is not associated with abdominal surgery, however, the importance of these bacteria cannot be ignored [19]. We isolated *B. pyogenes* from SSI after amputation due to chronic inflammation and necrosis of the toe in a patient with diabetes mellitus. *B. pyogenes* was cultured from the sample collected during wound purifying, and osteomyelitis was demonstrated by radiological examination.

*Bacteroides* spp. are characterized by the highest antibiotic resistance among anaerobes visibly increasing for over a decade [2, 20]. *B. pyogenes* isolated from human or animals has previously been shown to be highly susceptible to penicillin (lack of beta-lactamase production). This distinguishes it from other species of the genus *Bacteroides* [9, 13, 17]*.* However, in the presented study, seven isolates were resistant to penicillin. Due to the limited number of analyzed strains, it is difficult to accurately assess the drug resistance profile of *B. pyogenes*, which is a significant limitation of this work.

## Conclusions

Laboratory diagnosis of anaerobic infections is still a challenge. The reliability of the microbiological test result significantly depends on pre-laboratory procedures and access to molecular methods that are effective in identifying anaerobes. Currently, clinical laboratories have better access to modern, accurate methods. Consequently, new or previously unassociated with human infections species of bacteria are identified. One of them is *B. pyogenes* which, causes serious and chronic infections which predicts long-term hospitalization and intravenous therapy*.* Its pathogenic potential and resistance to antibiotics should be further observed.
